# Context matters: athletes’ perception of dopers’ values, actions and vulnerabilities

**DOI:** 10.3389/fspor.2023.1229679

**Published:** 2023-12-15

**Authors:** Annalena Veltmaat, Dennis Dreiskämper, Sebastian Brueckner, Dmitriy Bondarev, Andrew Heyes, Vassilis Barkoukis, Anne-Marie Elbe, Lambros Lazuras, Alessandra De Maria, Arnaldo Zelli, Andrea Petróczi

**Affiliations:** ^1^ Department of Sport and Exercise Psychology, Institute of Sport and Exercise Sciences, University of Münster, Münster, Germany; ^2^ Willibald Gebhardt Research Institute, University of Münster, Münster, Germany; ^3^ Institute of Medicine and Life Sciences, Immanuel Kant Baltic Federal University, Kaliningrad, Russia; ^4^ Faculty of Sport and Health Sciences, University of Jyväskylä, Jyväskylä, Finland; ^5^ Data and Marketing Analytics Department, SSM Sisä-Suomi Oy, Jyväskylä, Finland; ^6^ School of Life Sciences, Pharmacy and Chemistry, Faculty of Health, Science, Social Care and Education, Kingston University, London, United Kingdom; ^7^ Carnegie School of Sport, Leeds Beckett University, Leeds, United Kingdom; ^8^ Department of Physical Education and Sport Science, Aristotle University of Thessaloniki, Thessaloniki, Greece; ^9^ Department of Sport Psychology, Faculty of Sport Science, University of Leipzig, Leipzig, Germany; ^10^ School of Sport and Exercise Science, University of Lincoln, Lincoln, United Kingdom; ^11^ Department of Movement, Human, and Health Sciences, University of Rome “Foro Italico”, Rome, Italy; ^12^ Faculty of Education & Psychology, ELTE Eötvös Loránd University, Budapest, Hungary

**Keywords:** doping, qualitative inquiry, values, vulnerability, elite athlete, focus group, interview, risk factors

## Abstract

**Background:**

Although athletes seem to hold uniform views towards non-dopers, their perception of dopers is more nuanced, reflecting positive and negative attributes. Research also indicates that rarely a single factor can explain doping, but a host of reasons that intertwine. A holistic understanding of how values play a role in decisions in anti-doping and the elements that influence athletes’ doping vulnerability is timely and warranted.

**Methods:**

We recruited elite athletes from 13 countries representing 27 sports at a national or international level (*N* = 60) to participate as part of a larger research project. Data were collected via focus group interviews focusing on values, value priorities and perceptions about the role of values in doping as a phenomenon and in dopers’ actions. Data were analysed using iterative thematic analysis.

**Results:**

Three themes were identified: (1) athletes’ personal stance on doping, (2) dopers in the eyes of the anti-doping-compliant athletes, and (3) doping vulnerability is a balance. Athletes in this study strongly opposed doping but showed empathy and understanding toward athletes who doped under certain circumstances. Furthermore, athletes believed that “clean” and “doping” athletes are not always distinguished by the values they hold, leading to the realisation that all athletes can be vulnerable to doping at some point. This vulnerability is a balance between risks and protective factors in a complex interaction between environmental, personal, and situational influences. Each element (e.g., values, environment) can be a motivator or a barrier. Consequently, doping vulnerability is highly idiosyncratic and dynamic.

**Conclusion:**

If doping is not due to a lack of moral values but the consequences of combined risk factors that override the guiding function of values, then doping can happen to anyone, “good” athletes included. Developers and facilitators of anti-doping education programmes are advised to embrace this important aspect. The results also contribute to developing the doping vulnerability concept as a balance between risks and protective factors and draw attention to the clean athlete vulnerability, which is rooted in the combination of strategic performance enhancement via non-prohibited means, their exposure to anti-doping requirements and the constant high level of suspicion that surrounds them.

## 1. Introduction

Historically, anti-doping efforts have focused on detection-based deterrence through doping controls and sanctions (1, 2). However, despite the increased number of control tests, available prevalence rates indicate that doping continues to be a significant concern (3) and remains a contentious issue in sports research (4). In addition to the direct consequences faced by athletes who engage in doping, doping behaviour is also associated with unintended harmful consequences that directly or indirectly impact clean competitors who must coexist with those using performance-enhancing substances (5).

The recent sector-wide study among anti-doping stakeholders, which developed a research agenda for the next 10 years to inform the World Anti-Doping Agency's Social Science research programme (6), emphasised the importance of fostering environments and policies that promote clean sport (4). In this context, clean sport can be broadly understood as a culture where athletes adhere to principles of fairness, integrity, respect, and ethical conduct, free from any form of cheating, including the use of performance-enhancing drugs or methods, while upholding the values and spirit of sport (7).

Protecting clean athletes and ensuring a level playing field are fundamental principles of anti-doping policy, as clean athletes are the primary beneficiaries of these measures (5, 8). However, the prevailing emphasis on moralistic reasoning in discussions about doping in sports leads to a dichotomous view, labelling athletes who use performance-enhancing drugs as “bad” and those who do not as “good.” This oversimplified perspective not only narrows the focus on catching cheaters but also limits the understanding of vulnerability factors. Further, it promotes anti-doping approaches that prioritise punishment and deterrence over addressing underlying drivers of doping behaviour, and stigmatises athletes deemed “bad” for engaging in doping, obstructing their reintegration into clean sport (9, 10). Consequently, this “one size fits all” black-and-white view may result in limited success in reducing doping. Fostering a more nuanced approach that reflects the complex doping dynamics can improve our understanding of doping in sports and, accordingly, inform relevant anti-doping policies and practices.

This would highlight a recent shift from viewing athletes as inevitably engaging in doping unless prevented to recognising that most athletes genuinely desire clean competition (4). It also emphasises the importance of not neglecting clean athletes just because they were previously deemed non-problematic cases. Instead, their needs should be acknowledged, and appropriate support should be developed within the anti-doping system. Therefore, the perspectives of athletes who identify with a clean sports identity can help reveal factors that contribute to their resilience against doping. Additionally, it can help identify potential vulnerabilities, such as perceived pressures, motivations, and rationalisations that might lead them to consider doping, even if they currently adhere to clean sports values. Examining these aspects provides an understanding of the psychological factors of doping avoidance and helps recognise potential weaknesses or challenges that clean athletes may encounter in maintaining their commitment to clean sport.

### 1.1. Values in doping: a relevant but insufficient explanation

Recognising the importance of promoting clean sport, the World Anti-Doping Agency (WADA) introduced mandatory values-based education (VBE) as part of anti-doping efforts. The goal of this education is to cultivate personal values that protect the spirit of sport and create a clean sporting environment (11). More specifically, WADA's International Standard for Education defines VBE as “delivering activities that emphasise the development of an individual's personal values and principles. It builds the learner's capacity to make decisions to behave ethically.” [(11), p. 9]. Given that values guide people's actions across various situations (12), researchers have acknowledged their significance in understanding doping behaviour [e.g., (13–16)].

While research has demonstrated that moral values are positively associated with competing clean (15) and negatively associated with doping use [e.g., (16)], the question of whether dopers and clean athletes differ significantly in their value systems or if other factors contribute to vulnerability to doping remain to be elucidated. In fact, stereotypical perceptions of dopers as immoral actors prioritising winning over sports integrity are misleading and do not reflect the truth of athletes’ thought processes (9, 17). In a study conducted by Chantal et al. (18), participants reported a negative social image of a fictional elite athlete using anabolic steroids, yet subsequent research did not confirm this perception. According to their findings, participants associate steroid users with lower levels of self-determined motivation, a higher tendency for reactive aggression, and weaker sportspersonship behaviour compared to non-users. In contrast, other studies have shown that athletes’ perceptions of dopers are not solely negative and include favourable traits like confidence, motivation, and commitment (19). In sum, research has failed to find significant differences between doping users and clean athletes in terms of values and sportsmanship (20).

Values alone do not fully explain an athlete's decision-making process regarding doping. Contextual factors and coping strategies, such as normalisation or moral disengagement, can also play a role (13, 21–23). Additionally, one can view doping as a deliberate and rational decision supported by justifiable reasons (13, 24). This perspective frames doping as a functional behaviour (i.e., performance enhancement) rather than a deviant act (9). Consistent with this view, WADA's Athlete Vulnerabilities Research Project (25) emphasises that athletes can be at risk of doping at different stages of their careers due to a variety of factors. This vulnerability is not exclusive to those with dark personalities or “wrong” values, as all athletes could potentially engage in doping when faced with certain situations, despite their prior intentions or values [e.g., (10, 17, 25)].

### 1.2. Vulnerability and deterrent factors in doping

The concept of vulnerability in relation to doping emphasises the multi-dimensional nature of this issue, showing that it is not solely an individual's personal attributes or circumstances that make one vulnerable to doping but a complex interplay of various factors, including cultural, economic, and social influences. Based on literature precedence, the life-cycle model (26) identified certain psychological traits linked to an individual's personality that can make athletes more susceptible to doping. These “vulnerability factors” can include an athlete's propensity for risk-taking or sensation-seeking, their self-esteem, beliefs about doping, or susceptibility to peer pressure. Subsequent research has explored whether certain traits can form a dopogenic personality (27). The term “dopogenic” describes the collective impact of environmental and structural factors, opportunities and circumstances that foster anti-doping rule violations. Thus, it emphasises the interaction between athletes, their social surroundings, and the structures that guide their lifestyles and decision-making (28). With respect to personality, it refers to individuals who, by virtue of their inherent personality traits, may find it more acceptable to embrace questionable actions as they strive for success, thus leading them to hold a favourable view of doping (27). For example, research by Nicholls et al. (29, 30) found that the three traits of the Dark Triad - Machiavellianism, narcissism, and psychopathy - were all linked with more positive attitudes towards doping and cheating. These traits were identified as risk factors, predisposing athletes to adopt behaviours like doping more readily. Zhang and Boardley (31) also suggest paying close attention to vulnerable narcissism as a risk factor for doping. Perfectionism, specifically evaluative concerns perfectionism (concerns over mistakes, feelings of discrepancy between expectations and performance, fear of negative social evaluation, negative reactions to imperfection and parental pressure to be perfect), was also found to be a significant predictor of positive attitudes towards doping (32–34). However, other factors like self-oriented striving for perfection (35) were associated with more negative attitudes towards doping, suggesting they could serve as protective factors.

In addition to these personality-based vulnerability factors, there are systemic factors tied to an athlete's career progression, such as the motivational climate, perceived fairness of anti-doping strategies, authority structure, and the performance enhancement culture in teams and the broader athletic community. There are also factors that could potentially discourage athletes from doping. These deterrents can include things like the threat of sanctions, cultural or religious norms, personal moral values, pressure from family or friends, a stable sense of self-esteem, a low tendency to take risks, and concerns about health (26, 36). Accordingly, as values may influence an individual's decision to engage in doping, they represent only one small piece of the larger puzzle of doping complexity. Finally, situational factors, like the presence of role models and significant others, the nature of peer interactions, as well as athletes’ awareness of and access to non-doping alternatives, can change the relationship between an athlete's personal characteristics and the systemic factors they encounter. The importance of the context in understanding athletes’ decisions about doping has been highlighted in various empirical, mostly qualitative, research [e.g., (8, 17, 37, 38)]. In addition to the major groups of vulnerability factors in doping, both the life-cycle-vulnerability model (26) and the IMDB (9) recognise that influential factors vary across the various stages of athletic development. Notably, while the IMDB builds on the life-cycle model, both models have complimentary yet different premises. Although both models encompass doping as goal-oriented behaviour, the IMDB focuses on the progressive and situated nature of athletes’ decision-making processes concerning doping over the different stages of their sporting careers. In contrast, the life-cycle model places its emphasis on the expectancy-outcome feedback as a determinant for re-engaging or disengaging in doping and thus operates with a shorter timeframe. Understanding that certain athletes might be more vulnerable at specific career stages compared to others can provide a more nuanced view of how vulnerability to doping changes throughout an athlete's career, highlighting the need for further research into how these factors evolve over time.

A number of subsequent studies confirm this theoretical framework, suggesting that both internal and external factors combine to determine an athlete's vulnerability to doping. Kegelaer et al. (39) identified multi-level incentives, including athletic (performance improvement, injury recovery), psychological (perceived pressure, mental benefits), psychosocial (direct influencers, media pressure, social status improvement), financial gains, and policy-related (ineffective policies) factors. In contrast, the most important deterrent at the psychological level was morality coupled with critical thinking. Furthermore, anticipated guilt and shame upon testing positive for doping were reported as primary protective factors by female triathletes (40). Factors like poor supervision and precarious professional athlete conditions can increase doping susceptibility (41). Nicholls et al. (42) categorised athletes into different groups based on their doping susceptibility, identifying those with low self-esteem, high influence from their reference group, low trust in doping tests’ legitimacy, and high willingness to cheat as more at risk. Life and career events, such as the transition from youth to elite senior level, were highlighted as potential catalysts for doping due to increased internal and external pressures (39).

Overall, the current state of research emphasises the need to frame the knowledge about internal attributes (e.g., values and personality) within the larger contextual structures that influence an individual to gain a nuanced understanding of athletes’ vulnerability to illicit performance enhancement.

### 1.3. Research aims

The present work draws upon the findings of previous research and the life-cycle model of performance enhancement. Firstly, this study aims to challenge the simplistic dichotomy of “good vs. bad athletes” by exploring how athletes perceive the values and value systems of those who engage in doping. While acknowledging the importance of values in this context, the study also underscores that such considerations alone are insufficient to comprehend the complexity and diversity of doping behaviour. With this in mind, the second objective of the study is to discern not only the motivators, or vulnerability factors, behind doping but also to uncover factors that can act as a safeguard against it. Specifically, we aimed to answer the following questions: (a) What do athletes think of doping and dopers?, (b) What values do athletes link to doping and clean sport behaviour?, and (c) What factors can make an athlete vulnerable to doping, and what factors can protect against these threats?

## 2. Methods

### 2.1. Research context and positioning

The questions explored in this study form part of the wider “Sense-Making in Anti-doping Reasoning Training” (SMART) project. This 3-year international initiative aimed to create case-based anti-doping educational materials to enhance athletes’ sense-making and decision-making skills in complex ethical scenarios. Developed through a partnership between anti-doping researchers from Germany, Greece, Italy, Russia, and the UK, the project facilitated focus group discussions with elite athletes. These discussions centred on two key topics: (1) the evolution, prioritisation, and management of athletes’ personal values across varying situational contexts, and (2) athletes’ perceptions of doping and those who dope, particularly regarding the values and value systems of dopers. Results from the first topic are published in Petróczi et al. (43).

We analysed the data from the second, doping-specific topic from a pragmatist position, which epistemologically allowed us to commit to giving voice to athletes about concrete, practical and real-life issues (e.g., views on doping and dopers, values in decisions about doping and cheating, and vulnerabilities) without engaging in unhelpful debate about the nature of truth and reality, or prioritising how knowledge is created over why it is important to explore and share it, and how it can be done (44). As researchers in this study, we firmly position ourselves to respect that there is no one truth or reality in individual thoughts, feelings, and experiences, nor is one’s perceived reality more valid than that of others. Importantly, we did not intend to, nor claim to, establish what values drive doping or make athletes vulnerable to doping *per se*, but show how athletes who experience doping in their sporting environment *perceive* these issues. At the same time, we are aware of our influence and the interplay between athletes’ experiential knowledge and our more theory-informed subject expertise in the process of extracting and summarising data into themes. From the pragmatic perspective, we see this as an advantage for developing practically relevant, problem-focused themes that are useful for anti-doping policymakers and educators, whilst preserving the authentic thoughts and views that athletes shared with us. This pragmatic approach allowed us to keep the focus on the empirical issues at hand, namely how values influence decisions in sport and anti-doping; and explore the roles athletes’ values play in decisions about cheating, doping, or following clean sport principles. Underpinned by pragmatism, we could use a combination of theories, concepts, and employ an iterative approach to thematic analysis to capture the complexity of the issues at hand whilst benefitting from the cumulated knowledge and expertise of the research team, including those who were not directly involved in the coding process and the initial theme generation. In our analysis, we adhered to the core methodological principles that underlie a pragmatic approach to inquiry namely, we kept an emphasis on generating actionable knowledge (e.g., offering insights for anti-doping policies and education), considered the study as an iterative experiential process in which we recognised the interconnectedness between the athletes’ authentic experiences and views, our expert knowledge and the context where the new knowledge can be utilised.

In keeping with the pragmatist approach, in this study, we use a narrow definition of doping, which is the conscious and goal-oriented use of prohibited substances and/or methods. We are aware that this definition is significantly more restrictive than the definition set by the World Anti-Doping Code (45), which does not require intention, only the presence of prohibited substances or evidence for the use of prohibited methods. To differentiate between intentional doping and inadvertent rule violation due to lack of vigilance, poor labelling or contaminated products, we refer to the latter as inadvertent anti-doping rule violation. We opted for the narrow definition of doping, which places emphasis on *decisions about doping behaviour* (which is a conscious choice) because we were interested in how athletes (at the individual level) see doping, what values they connect to doping and how they experience protective and risks factors within the realm of athlete vulnerability, as opposed to how the anti-doping *system* and its organisations define, legislate, detect, sanction or prevent doping in their quest of protecting clean sport and athletes’ rights to clean sport. The need for closing the gap between the two levels has been noted previously [e.g., (7, 46)].

### 2.2. Study design and participants

The study utilised criterion-based intentional sampling (47) and selected 60 elite athletes (37 male, 23 female) aged 17–48 (Mean = 26.1; SD = 7.02) that met the inclusion criteria of routine competition at the high level in their sport and subject to WADA doping testing standards (see Supplementary Material S1). These athletes, either currently active (*n* = 47) or recently retired (*n* = 13), represented 27 different sports, with Athletics (*n *= 16), Soccer (*n* = 4), Handball (*n* = 3), Sailing (*n* = 3), and Weightlifting (*n *= 3) being the most common. All had competed at national or international levels, including five Olympians/Paralympians and 29 European or World championship competitors (nine with medals). Notably, despite their high-level competition, 25 athletes had never been tested for doping, and 23 had received no formal anti-doping education.

We did not specifically ask the participating athletes about their involvement in doping. It was up to the athletes to reveal as much or as little about their personal experience as they felt comfortable with. However, from the focus group discussions, we assume that at the time of the data collection, all participants in this study were anti-doping compliant (clean) athletes. Nonetheless, they were still confronted with prohibited performance enhancement, either through direct contact with doping users, anti-doping interventions, the media, or in their sport environment.

### 2.3. Procedure

After obtaining institutional ethical approval, the research team conducted a two-phase data collection. In the first phase, international partners from Greece, the UK, Germany, Russia, and Italy recruited athletes who met the study criteria through national anti-doping agencies and personal contacts. Six national focus groups were conducted via online platforms in the UK (*n* = 7), Greece (*n* = 2 × 5), Germany (*n* = 5), Italy (*n* = 6), and Russia (*n* = 7), with discussions held in the participants’ native languages. The focus groups, hosted by one of the listed authors, lasted between 63 and 120 min, depending on the flow of conversation (M = 94.33, SD = 19.04). In the second phase, we conducted seven international focus groups in English (*n* = 25), each lasting between 84 and 109 min. These discussions were held on a secure videoconferencing platform and led by an experienced qualitative researcher and an athlete researcher. All discussions were recorded, transcribed verbatim, and translated into English where necessary for analysis. To ensure the anonymity of the participants, we used acronyms (Athlete 1 - Athlete 60) to present the results and redacted any information that could identify athletes.

### 2.4. Data collection

In line with the principles of generic qualitative inquiry (48), a semi-structured focus group guide was developed by the researchers to explore how athletes interpret and make sense of their values across different situations and times. Specifically, the focus group guide was divided into four main sections. Sections one to three focused on athletes’ personal values and value systems. In section four, which generated data for the present study, we asked the participants about the role of values in the context of doping and performance enhancement.

The relevant interview questions were as follows:

1. Do you think athletes who dope have different values? Or do they change their values priorities? (Prompts: Do you think they believe doping is okay and within their values?)
2. What do you think changes in their situation that lead to doping? (Prompts: Injury? Illness? Poor form? Poor progress? Social pressure?)
3. Why do you think doping is generally socially unacceptable? Do you think society's views on doping will change over time?

Focus group discussions were moderated by an academic researcher with qualitative research experience and co-moderated by an athlete or retired athlete with relevant research training. Participating athletes were encouraged to speak freely and have a discussion among themselves as opposed to responding to the moderators’ questions. Moderators ensured that all athletes were given the opportunity to speak. Although athletes were asked, as part of their consent to participate, to keep the information they may learn confidential, athletes were reminded at the start of the session to be mindful of sharing confidential information about themselves or about other athletes.

National focus groups were conducted in the respective native languages; international focus groups were conducted in English. Due to recruitment via personal contacts, some athletes (especially in national focus groups) knew each other if they were from the same sport. All focus groups were done via an online platform due to social distancing restrictions during the SARS-CoV-2 pandemic. Discussions were audio or video-recorded and transcribed (or translated and transcribed in English) verbatim.

### 2.5. Data analysis

The data were analysed using an iterative codebook analysis inspired by (49–51). We applied codebook analysis as conceptualised and defined in Braun and Clarke (49, 50) for two reasons: Firstly, codebook analysis is well suited for applied research. Secondly, we were motivated to stay close to what athletes said, to give them a voice via presenting themes as topic summaries while offering research context for practical relevance. Braun and Clarke's (49) codebook analyses afforded us to do so. In addition, we opted for an iterative approach (51) because it is philosophically based on pragmatism and thus aligns well with our applied problem-focused research aims. This approach allowed us to develop the themes as early as possible through the subject knowledge of the authors, who collectively have over 100 years of research experience in doping and anti-doping, and to engage in a continuous revision of the results through writing this manuscript to generate the final set of themes.

We followed Braun and Clarke's (52) six-step approach to thematic analysis, which provides a framework for identifying, analysing, and reporting patterns (themes) within data. Specifically, the study employed a thorough familiarisation process with the data, during which initial codes were generated. These codes represented the most basic elements of the raw data that appeared noteworthy. Both latent and semantic aspects of the data were considered during the coding process. We conducted the data analysis in NVivo v12. Following coding, subthemes were identified. These subthemes represented smaller patterns or categories within the data derived from the initial codes. They provided a more detailed and nuanced segmentation of the data, enabling a more comprehensive reflection of the participants’ experiences and opinions.

Next, these subthemes were analysed for broader patterns across the data. These patterns constituted the main themes of the analysis. This stage of analysis was iterative, involving a constant movement between the entire data set, the coded extracts, and the analysis of the themes themselves. These themes were then reviewed against the dataset, ensuring they accurately represented the summaries extracted from the data. During this review, themes were refined, which involved splitting, combining, or discarding some to accurately summarise the data.

Finally, the main themes were identified and labelled, reflecting the larger patterns and important aspects of the data. We created a thematic map to graphically illustrate the relationships between the themes and subthemes, providing a visual representation of the findings. Throughout this process, the emphasis was on summarising the data through identifying repeated patterns of meaning rather than interpreting or providing deeper reflections on it. The authors collaboratively agreed on the final names of the themes and subthemes during the write-up of the results (see Supplementary Material 2).

**Figure 1:**
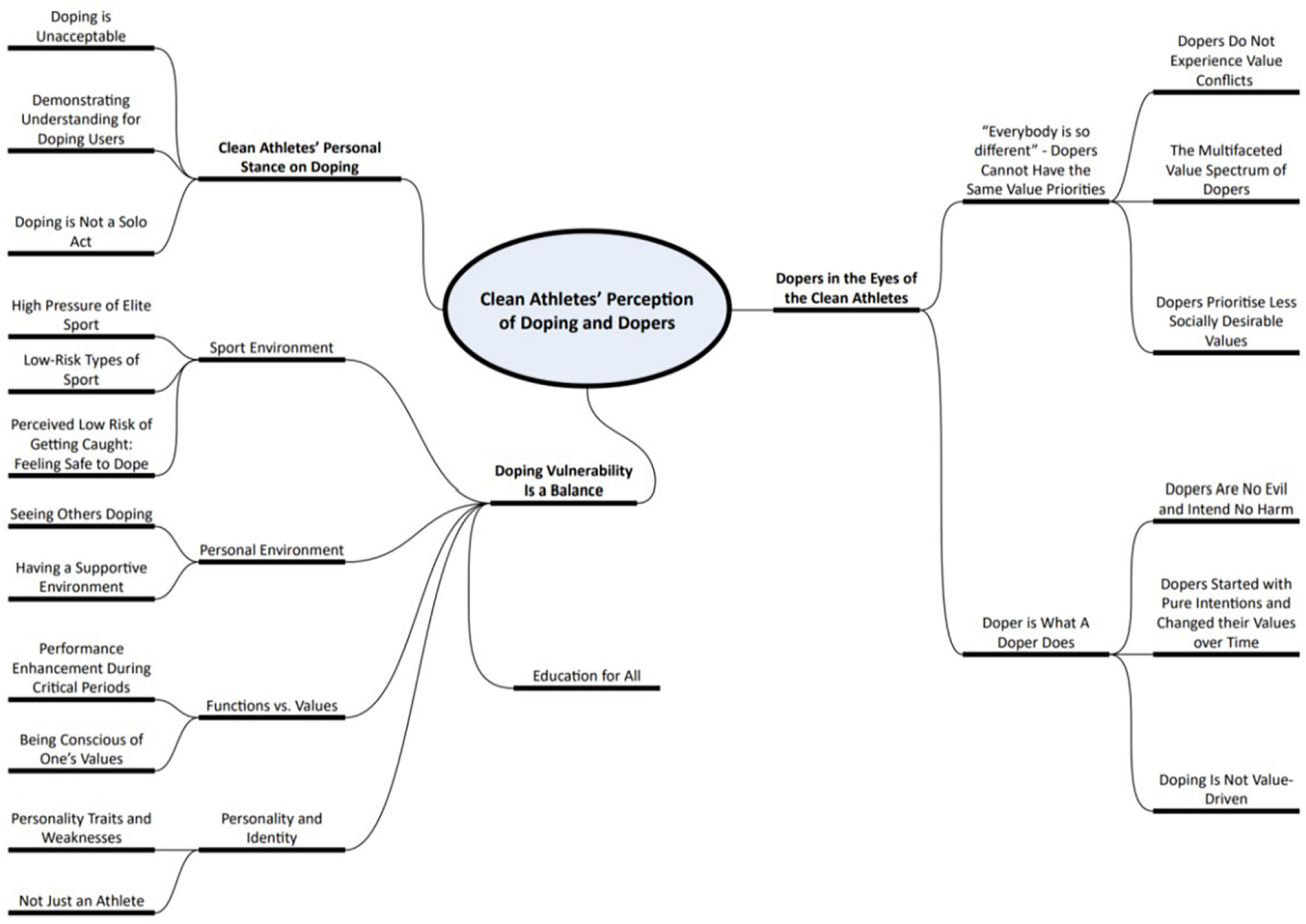
Structure of themes and subthemes.

At the writing stage, the initial themes were revised and finalised for the final visual map (Figure 1), which reflects the interaction between our knowledge, expertise, and initial and revised beliefs about values and vulnerabilities. This process undoubtedly benefitted from multiple factors, which include the involvement of anti-doping experts (AP, A-ME, AZ, DB, DD, VB) and an athlete researcher with qualitative research experience (AH).

### 2.6. Research quality

#### 2.6.1. Methodological integrity

Rooted in a pragmatist approach, the intention to extract meaningful, actionable knowledge from athletes’ lived experiences and perceptions surrounding doping drove our study. Instead of aiming for a singular objective “truth”, our goal was to illuminate the multifaceted, complex realities athletes navigate. Drawing from a relativist stance (53), our pragmatist underpinning led us to adopt a qualitative method, emphasising the subjective, lived experiences of athletes. This choice stems from our conviction that comprehending these subjective nuances is pivotal for practically addressing our research queries and contributing meaningfully to the anti-doping discourse.

#### 2.6.2. Trustworthiness and rigour

Opting for trustworthiness over reliability underscores our focus on the authenticity of athletes’ voices and their perspectives. The codebook approach (49) guided our analysis, anchoring our interpretations in the genuine narratives of participants. Ensuring rigorous analysis, we employed several measures:

First, our continuous *reflexive engagement* throughout the thematic analysis was evident as we used reflexive journals to document emerging biases, thoughts, and preconceptions. Second, the *collaborative theme development* began with initial themes identified and discussed with the authors AH and AP. AP played a crucial role in refining these themes, facilitating their grouping, labelling, and other adjustments. Third, *feedback integration* was a systematic process where we assimilated feedback from the authors into our analysis. Regular consultations fortified our interpretations. Fourth, *cross-validation and consensus-building* were paramount. Themes underwent mutual validation among team members, culminating in a consensus after rigorous discussion. This process ensured that our findings were not the product of a singular viewpoint but reflected a collaborative understanding. Fifth, *the diversity within our research team and our commitment* to the cause played a significant role. We made certain that our findings transcended individual biases and authentically represented athletes’ realities.

#### 2.6.3. Contribution to knowledge and debate

In alignment with the advocacy of Sparkes and Smith (53) and Smith and McGannon (54), we embraced a nuanced and open-ended research approach. Guided by their emphasis on authenticity, context, and researcher reflexivity, our study sheds light on the multifaceted perceptions athletes hold about doping. Rather than relying on fixed criteria or adhering to singular narratives, we have integrated the fluidity and flexibility they advocate in evaluating qualitative work. Consequently, our findings challenge monolithic viewpoints and introduce intricate dimensions to the doping discourse. Through this, we hope not only to enhance anti-doping policies but also to invigorate discussions around sports ethics and practices.

#### 2.6.4. Limitations and positioning

It is essential to note that while we sought athletes’ views on doping, we did not aim to make definitive claims about the values of dopers or establish objective facts about doping practices. Our participants’ insights, though valuable, are influenced by a myriad of factors and should be interpreted as individual perceptions rather than overarching truths. That is not a limitation but a clear positioning of our study within the relativist criteriology framework (53).

## 3. Results

Applying the described thematic data analysis approach, we identified three themes: (1) athletes’ personal stance on doping, (2) dopers in the eyes of the anti-doping compliant athletes, and (3) doping vulnerability is a balance between risks and protective factors. Figure 1 shows the detailed structure of the three themes and their subthemes.

### 3.1. Athletes’ personal stance on doping

The first main theme we developed was the participants’ personal stance on doping. Sub-themes and topics captured can help better understand why these athletes refused to engage in doping and to classify their assumptions about the value systems of athletes who dope. Within this theme, three sub-themes emerged: (1) Doping is Unacceptable, (2) Demonstrating Understanding for Doping Users, and (3) Doping is Not a Solo Act.

#### 3.1.1. Doping is unacceptable

Reflecting on their reasons for rejecting doping, participants primarily deemed doping as morally unacceptable, arguing it contradicts the core values of sport. They viewed doping as a form of cheating that disrupts equality among athletes and negatively impacts others, distinguishing it from self-focused modifications, such as altering one's appearance.

Their decisions to avoid doping were deeply intertwined with their personal values. Violating these principles, they believed, would lead to feelings of guilt, shame, and dissatisfaction with their performance. One participant (Athlete 58) indicated: “The idea that I know that I'm cheating would be so frustrating…I don't think I would allow myself to do that.”

In contrast to the anticipated negative emotions associated with doping, some athletes revealed the positive feelings derived from staying clean, even during challenging periods in their careers. One athlete emphasised the pride and sense of responsibility she derives from being a clean athlete and how it influences her role as a mentor, stating, “When life hits you hard, and there is an easy route, don't take it…it's something that I can be proud of.” (Athlete 42)

#### 3.1.2. Demonstrating understanding for doping users

Although almost all participants explicitly stated that they view doping as wrong and distanced themselves from those who engage in this practice, some statements indicated a certain level of understanding of the doping decision of others. They acknowledged that one's choices could change based on evolving personal circumstances and challenges. As Athlete 58 observed:

From this point of view and with this mindset, with these circumstances, I would always say definitely not. But at the same time, who knows what might happen, what may happen to me like in two years’ time, for example, what could be my life circumstance […] I've witnessed so many people changing their core values not because they have nothing else to do, but because in their belief that was the only thing, they actually could do to change their life circumstances without thinking of the consequences.

These comments suggest that values can be fluid, changing based on the severity of influential factors. The athletes’ curiosity to understand the value priorities of others and how they might arrive at a decision to use doping under certain conditions supported this idea further. These observations laid the groundwork for a later analysis of risk factors that could lead athletes to adjust or abandon their value priorities during the decision-making process.

#### 3.1.3. Doping is not a solo act

Although the WADA code (2021b) unequivocally states that it is the athlete's duty to ensure that they do not commit anti-doping rule violations, some participants highlighted that doping is not solely rooted in an individual athlete's decisions and behaviours. Hence, they assumed that it was not the athletes’ initiative to dope in many cases but that other people influenced their behaviour. Athlete 15 summarised this in a statement:

In my opinion, what also matters is the people who are around you…I think that the issue of doping is not only about a single choice of an athlete…but also a general mentality in the sport context, in the society at large, in the people.

Participants also noted that someone's susceptibility to doping is often based on their influenceability by these authority figures rather than personal failings. They are particularly vulnerable in emotionally intense situations or under significant performance pressure. Athlete 21 highlighted the role of coaches, stating: “I think the coach plays a very important role…if the coach thinks only of winning…this is transmitted to the athletes and their behaviour changes dramatically.”

On a larger scale, some participants noted systematic pressures in certain countries where athletes may feel they have no choice but to dope. Athlete 55 stated: “I think…the system behind is a big part of that…when you see some countries, they are pretty big, and I think that leads sometimes when we talk about doping maybe they [dopers] have not really other options. You do it, or you're out.”

### 3.2. Dopers in the eyes of the athletes

To examine if athletes perceive differences between them and dopers and to subsequently explore if unfortunate circumstances cause deviating value priorities, the interviewer asked participants to reflect on the value systems of dopers. Participants highlighted different priorities on the one hand but also clarified that rule breakers are not necessarily evil people. Some even pointed out that they do not associate (anti-)doping with values at all. Within this second main theme, the following sub-themes emerged: (1) “Everybody is so different” – Dopers Cannot Have the Same Value Priorities; (2) A Doper is What a Doper Does. Each subtheme contains multiple categories that reflect athletes’ perception of dopers.

#### 3.2.1. “Everybody is so different”—dopers cannot have the same value priorities

Some athletes in the group felt that those who use prohibited substances or methods are inherently different from athletes who are committed to training and competing within the rules and that this difference is value- or personality-driven.

##### 3.2.1.1. The multifaceted value spectrum of dopers

The participants used the fact that doping cases still exist as the rational basis for their assumption that not all athletes share the same value systems. Hence, according to them, dopers cannot prioritise fairness and respect to the same extent as clean athletes since these values conflict with cheating behaviour. In their understanding, dopers and non-dopers having the same values yet exhibiting different behaviours was incongruous. However, they also acknowledged the heterogeneity among athletes who use doping, making it difficult to generalise their value systems. As Athlete 45 pointed out: “But what their integral values are, I wouldn't really know because everybody is a product of their environment, and so yeah, everybody is so different.”

They recognised that individual backgrounds, motives, and the broader social context could shape an athlete's value system and influence their attitudes towards doping. Athlete 58 further elaborated:

Especially in individual sports, we are completely different, there are different values in each and every one of us, and basically, I think that depending on the motive and your background and what actually formed you as a person when you were a little kid […] like there are people who maybe feel like having a doping case is okay as long as it's hidden but it will help them succeed.

In addition to these personal factors, participants also identified cultural differences, suggesting that values might vary between countries, with distinct notions of fairness and correctness across different regions, particularly between Western nations and less developed regions like Africa.

##### 3.2.1.2. Dopers prioritise less socially desirable values

Participants perceived a divergence in value priorities between clean athletes and those who use doping. They hypothesised that athletes who engage in doping tend to prioritise values related to winning, fame, and reputation over social values such as fairness and respect.

According to the participants, athletes who dope might place performance and financial gain above moral considerations, as suggested by Athlete 4: “[…] for them it's simply about performance, and it's about making money and the morals behind it are not so important. So for them, it's not about whether the competition is fair.”

Furthermore, they speculated that dopers might be more inclined to sacrifice their health and personal values for the sake of publicity and acclaim, as outlined by Athlete 24: “Some athletes might prioritise publicity and all these things which famous athletes have. They might prioritise this over their health, and along with the urge from some people around them, they will use doping.”

This implies a fundamental difference in the value systems of clean athletes and those who engage in doping. While athletes in this study underscored the importance of integrity and prioritised their values over performance, they suspected that dopers tended to prioritise performance over values.

##### 3.2.1.3. Dopers do not experience value conflicts

Athletes view the act of doping as a moral violation that would induce feelings of guilt and shame. However, they also express the belief that athletes who do engage in doping might not experience the same value conflict, pointing towards an attitude of indifference (a “don't-care” attitude) or a focus on personal benefits.

According to the athletes, doping athletes may not view their actions as problematic as they align with their priorities of success and excellence in performance. Athletes shared stories of clean athletes confronting dopers, only to be met with dismissive responses, indicating a lack of understanding or remorse for their actions from the dopers. Hence, the doping users did not show any understanding that their behaviour had negatively affected clean athletes.

Athletes suggested that dopers might justify their actions by viewing their methods as commonplace. In their minds, if everyone is doping, it is not immoral. This gradual shift towards accepting doping as a normal practice can make the moral conflict seem less severe. Athlete 20 explains: “[…] this [doping] is probably done gradually. Day by day, little by little, you slowly get into this thing obviously, and you have already accepted it, and it seems very small at some point.”

Moreover, it has been suggested that this process of justification and normalisation could be more prominent among athletes from less developed countries, where sporting success can be a means of escaping poverty. Athlete 4 shared this perspective:

[…] it's the case that in some parts of Africa that they know from the very beginning that they want to get out of poverty somehow and that the only thing that is going to lead to a better life is maybe earning money through running, and I think they also know from the very beginning that they are going have major difficulties achieving this if they don't take any drugs or if they don't begin doping right from the very start. And they actually have no conflict with their values at all because, for many of them, it's the most normal thing in the world. And for them, it's simply about performance, and it's about making money, and the morals behind it are not so important.

#### 3.2.2. A doper is what a doper does

This subtheme represents views that are opposite to the belief that athletes using prohibited means are inherently different (type of) people than clean athletes. Instead, the variance lies either in the situations in which they were tempted or pressured to use doping or in their individual responses to these temptations or pressures influenced by their personal circumstances. There is also a recognition that doping use is seldom a lonesome act. Even if others in the athlete environment do not play an active part in doping, they most likely have a certain level of knowledge of it and at least are turning a blind eye. Moreover, a local permissive culture may indicate a perception that doping is less risky as there exists a certain level of organisational support for individuals who tested positive, either during the sanctioning process or when athletes wish to return to competitive sport in some capacity (i.e., as an athlete, or as a coach) after a ban.

##### 3.2.2.1. Dopers are no evil and intend no harm

Although they disapprove of the behaviour of doping users, a remarkable number of participants argued that dopers are not necessarily fundamentally bad people. Instead, they believe that the stories and high-profile doping cases portrayed in the media are not an accurate representation of the individuals involved. Athlete 10 reflected on this:

I don't know any dopers, but I think the media likes to scapegoat and make out obviously they have big stories about various dopers […] I think it's a really dangerous thing to do because dopers are probably way more like you and me than people probably give them credit for.

In contrast to the myopic view that dopers are simply cheaters who willingly engage in prohibited practices at the expense of their fellow athletes, participants in the given sample argued that doping users do not primarily dope to harm others but rather that it is a consequence of unfortunate circumstances. In this regard, they frequently referred to the conditions of professional athletes from poorer countries. In line with that, Athlete 59 explained that the underlying motivation of African distance runners to dope is presumably not to cheat others but to improve the living conditions of their home area and families:

I think it's more a humanitarian issue than the choice to cheat […] I see it more from the perspective that they don't see it as they're doing wrong, they're probably trying to get themselves out of poverty and provide for a family or even a village.

##### 3.2.2.2. Dopers started with pure intentions and changed their values over time

The participants indicated that no athlete starts sports with the predetermined goal to dope. Athlete 29 remarked, “I think all athletes start sports with pure intentions. Nobody begins thinking about doping…this usually happens gradually.”

They hypothesised that while all athletes share similar values when they start, different circumstances can cause these values to change or get abandoned. Athlete 26 stated, “When you begin as an athlete, you share common values, health, enjoyment of your sport, and progression. However, somewhere along the way, some values might diverge.” Participants identified several factors that might cause this shift in values, including one's upbringing, daily interactions, coaching, and culture. Athlete 46 said, “The environment you grow up in, the people you communicate with daily, your coach, and your culture could influence a change in values and goals.”

Interestingly, some participants admitted that their own values could potentially change given certain circumstances. Athlete 57 commented, “There probably isn't a significant difference between me and those people. I believe there's a set of circumstances where my values could change to a point where they'd be unrecognisable to me.”

When reflecting on the exact circumstances that might provoke the value change, the participants frequently referred to external pressure as a potential reason. Hence, they explained that the high expectations of their close surroundings (e.g., parents or coaches) can pressure them to consider doping to achieve the desired results. They also noted that an athlete's personal motivation for success could drive changes in values.

##### 3.2.2.3. Doping is not value-driven

While discussing and comparing the value systems of dopers and clean athletes, some participants indicated that values might not be the decisive factors that explain doping behaviour. Instead, they argued that values do not necessarily find consideration in the decision-making processes of doping users, as described by Athlete 29: “I have rarely seen anyone seriously associate doping with values […] But in order to say there was a value in anti-doping, then everything seems to be quite ambiguous.” Likewise, Athlete 31 argued that anti-doping is not always value-driven by saying: “I also think that for some reason athletes cannot always believe that anti-doping is associated with values, yes, or that your values may run counter to the use of doping.” Beyond the assumption that values are simply not the main reasons why or why not athletes engage in doping practices, others highlighted that a process of normalisation and justification accompanies the decision to dope. Hence, according to the participants, dopers manage to disengage from their values by rationalising and defending the use of doping to themselves. Congruently, Athlete 16 explained:

These athletes may share values with those athletes who do not use doping, but at some point, they may however think that ‘the goal justifies all means’, and so, maybe it is an issue of character, or education or morality more than it is because of values.

### 3.3. Doping vulnerability is a balance

Even though we did not specifically ask athletes about vulnerability, much of the discussions revolved around factors, not just values, that may protect against or promote doping. Athletes’ doping vulnerability is presented as a balance between risk factors such as the perceived need for doping, external pressures, temptation, and conducive environments, and resilience factors such as personality traits, values, and proactive self-care against stressors.

The participants’ responses indicated that anti-doping measures are not just about an athlete's values, and one cannot reduce doping behaviour to mere character flaws. Instead, it is important to explore other reasons to understand which athletes are most vulnerable to doping. This informed the second objective of the study: Investigating other factors driving doping.

Risk factors are circumstances that could provoke a change in values or reasons why athletes might choose to dope. However, for effective anti-doping strategies, it is also crucial to identify protective factors that buffer against these risks.

From the analysis, five sub-themes emerged within this main theme. For each sub-theme—(1) the Sporting Environment, (2) Personal Environment, (3) Function vs Values, (4) Personality and Identity, and (5) Education for All—multiple categories emerged that could act as risk or protective factors depending on the conditions:

#### 3.3.1. Sport environment

##### 3.3.1.1. The high-pressure environment of the elite sport system

Participants identified the competitive environment and escalating pressure as they transition to the elite level as risk factors that could influence doping behaviour. They attributed this to external pressure, expectations to deliver exceptional performances, perceptions of others doping, and the perception of safety when using performance enhancers.

Upon becoming professional athletes, the participants experienced mounting pressure to excel, not just from personal ambitions but also their sporting environment. They suspected that athletes may resort to doping to meet these expectations. Economic factors add to the pressure; for many athletes, sport is their sole source of income, so the economic pressure to succeed can lead to doping temptations. Athlete 56 stated: “You've got all these external forces upon you…in most sports, only the very top…your top five or top ten are making a decent living…the pressures are just insane.”

The performance pressure has increased over the years as international competitions became more challenging, potentially leading some athletes to resort to doping. The participants criticised the lack of athlete protection due to temporary contracts and lack of labour rights. Athlete 51 highlighted:

You can say that you are a professional, but you don't have a contract, you don't have a salary…you are vulnerable…and if there is some situation where the athlete is more susceptible to doping because there is…you are not protected.

##### 3.3.1.2. Perceived low risk of getting caught: feeling safe to dope

Participants noted that athletes may be more tempted to use performance-enhancing substances if they believe the risk of getting caught is low. That could be due to infrequent testing in some competitions, as reported by Athlete 44: “Many athletes use substances because they know that in my tournaments we are not tested…that's why many just go into the drug store and buy [product redacted] for recovering and improving some physical features.”

Athletes may be especially tempted to dope in preparation for a competition if they believe the substances will quickly leave their system and thus be undetectable. Athlete 45 also suggested that athletes with lower rankings might feel safe from scrutiny:

I've only been tested once in competition. If my values were different, I might assume that I am low enough down the rankings that UKAD don't really care much about me…I could feel like maybe I could get away with doing something for two to three months because they are not going to look at me.

##### 3.3.1.3. My sport is not vulnerable, neither am I (low-risk types of sport)

Some participants noted that the risk of doping varies by sport, with those focused more on tactics or coordination seen as lower risk since doping is perceived as less beneficial. As Athlete 58, a sports shooter, put it: “I consider myself very lucky…my sport is definitely not in the group of so-called risky sports where we actually use so much of these [performance enhancers].” Athlete 27, an archer, echoed this sentiment, saying: “In my sport [archery], fortunately, there is no doping…there are mostly mental demands in the sport.”

Participants also highlighted the role of incentives provided by organisations. They suggested that the temptation to dope can increase when the stakes are high. Conversely, lowering monetary rewards could potentially reduce doping incentives. Athlete 45 gave an example from UK weightlifting: “There is no prize for winning at any level of weightlifting…I think the drive or what pulls an athlete to make those decisions is actually reduced quite a lot in weightlifting in the UK.”

#### 3.3.2. Personal environment

##### 3.3.2.1. Seeing others doping

Participants noted that athletes’ frequent interactions with teammates and competitors can shape their perception of doping. Athlete 38 pointed out a “sheep mentality” in their team, suggesting that observing teammates using doping without repercussions could likely lead to similar behaviour. This temptation is particularly strong when athletes see that doping provides a competitive edge, as Athlete 49 explained: “When you are in a competitive environment…if you see your teammates perform the best [by doping], then you'll see it as a goal to reach that level.”

Participants also suggested that this influence extends to competitors’ behaviour. They proposed that athletes are most likely to consider doping if they believe their competitors are doing it. Thus, the misconception that “everyone is using it” was identified as a significant risk factor for doping use.

##### 3.3.2.2. Having a supportive environment

The athletes’ immediate circle plays a crucial role in influencing their susceptibility to doping. This influence, contingent on the attitudes and values of close contacts, can serve as a protective factor. Athlete 51 noted the positive impact of being in a clean environment that emphasises the importance of anti-doping: “If you have an entourage where you talk about clean sport and you speak up about the importance of anti-doping, so you are more protected to that.”

Many athletes emphasised the influence of their coaches’ values on their behaviour in sports. As Athlete 7 pointed out, the coach's mindset encouraged them to maintain their integrity and focus less on performance pressure:

It was coaches that have installed in me from a young age that sport is about that integrity and it is how you conduct yourself which defines you rather than your performance level so yeah that's been the big one for me all the way along.

Support from family, especially irrespective of sports success, was also underscored. Furthermore, participants highlighted the importance of shared values with their professional partners, like sponsors. Athlete 22 appreciated that their sponsor upheld the same values, such as sportsmanship, which negated any pressure to resort to doping to meet expectations by saying: “Our sponsor is [name redacted] that represents the same values that we have, such as good sportsmanship and we feel no pressure to do extreme things just for satisfying anyone's expectations.”

#### 3.3.3 Functions vs values

##### 3.3.3.1. The functionality of doping: performance enhancement during critical periods

Success in professional sports, vital for securing contracts and sponsors, is a key driver for doping, according to many participants. They argued that athletes are likely to resort to prohibited substances when legal methods fail to enhance their performance. Some view doping as a functional way to navigate critical periods, such as performance stagnation or injuries. Accordingly, the fear of falling behind and the will to compete can cause an athlete to use doping, as indicated by Athlete 42:

Injuries play a big role, it's such a dark place when you have an injury because you know that you have a sustaining injury and you still want to compete […] I have seen so many people that fall for that because of injuries because they feel like if they don't take it [doping] I'm going to fall behind from my competitor, from other people, so therefore they feel like it's an obligation for them to maintain their stamina or maintain their performance by doping.

The temptation to use doping increases when athletes strive to regain their former performance level or are discontented with their current progress. Athlete 45 suggested that some may see doping as their only solution during these challenging times: “For some people […] it's their only outlet and they don't know any different.” The vulnerability to doping has been suggested to increase with age as older athletes feel a sense of urgency in their careers. Under these conditions, doping is perceived not as cheating but as a functional tool to restore performance capacity.

##### 3.3.3.2. Being conscious of one's (anti-doping) values

Participants identified that a conscious, clear anti-doping stance about anti-doping values is a protective factor. Athlete 19 explained how a deeply anchored set of values reduces the risk of doping:

I think that if there's a well-rooted set of values inside you, and it's well structured, that you start sport and you don't just think about the result, but you enjoy the process and the route and if you do that, it's somewhat harder to end up doping, I think.

Athlete 58 stressed a strict adherence to personal values: “I'm a completely black and white person it's like it's either black or white. So, if it doesn't match my values, it's not going to happen, I'm really very strict when it comes to that.”

Participants indicated that athletes with a strong anti-doping stance are less likely to be offered illicit substances, reducing the risk of violating principles. They emphasised the need for integrity, particularly in challenging situations like injuries or confrontations with athletes who do not share the same anti-doping values. They highlighted the need for mental strength and resilience to resist doping, suggesting that a focused mindset and self-confidence to handle setbacks are crucial.

#### 3.3.4. Personality and identity

##### 3.3.4.1. Internal attributes: personality and weaknesses

Participants argued that external factors may make them more vulnerable to doping. Athlete 56 indicated that external and internal pressures such as performance drops or competitor performance spikes could trigger doping: “It's when those external pressures and internal pressures ramp up so, you know, maybe you saw a drop in performance or competitors increasing its performance, that's usually the classic one.”

Participants noted that not all doping decisions are value-based; they often relate to personality and the ability to handle pressure. Athlete 26 considered doping susceptibility as primarily a character issue, influenced by upbringing and an individual's response to pressure during fragile stages:

Firstly, I would say that this is a character issue. Of course, it starts from home, but it's also the character of the child that if you approach him/her in a fragile age or moment, it will be the fault of the coach.

It was pointed out that each athlete's unique personality shapes their view on doping. Many participants linked doping use to personal weakness, indicating that an athlete's ability to resist doping depends on their strength in challenging moments, as stated by Athlete 16:

I am sure that there are tough moments, challenges, or temptations, but athletes are prepared to deal with them. For some, instead, it becomes an issue of personal weakness, and doping can happen.

The participants also noted that vulnerabilities become apparent during sensitive periods, such as during youth when athletes are more naïve and susceptible to influence or during performance drops, injuries, or illness, where resilience is crucial.

##### 3.3.4.2. Not just an athlete

Participants suggested that athletes’ dependence on sports for economic and social status can increase doping temptation. As Athlete 1 explained, athletes might resort to doping to maintain their social status or reputation when sports are their sole identity:

They [dopers] may only see themselves in the sport and might say, yeah, okay, but what can I possibly do if I can't do my sport anymore? So, I have to, or I have to stay with my sport no matter what the cost in order to, perhaps, maintain my social status, to further increase my reputation, or whatever. And that's why, yeah, I'm doping now, to push my performance further, maybe in order to keep up my circle of friends or whatever.

However, having an identity beyond sports was identified as a protective factor against doping. This includes not only having a social network outside of sports but also alternative career plans. Participants highlighted the benefits of having an academic degree as a fallback option, reducing reliance on sports and helping athletes manage performance declines during challenging times. Athlete 11 noted the benefits of having alternate career paths, contrasting it with athletes who may have fewer such opportunities and how that might push them towards doping:

I can't speak for other people, but I know that I'm quite fortunate in that if the door of hockey closes, I know that I have other opportunities and other career paths and we were discussing earlier with athletes from Kenya is that they don't have other opportunities and running is the only way to better their life, I could imagine that maybe that is an option.

#### 3.3.5. Education for all

Only a few participants emphasised the role of education in preventing doping. Those who did, claimed that education helped athletes reflect on their values and understand the consequences of doping. Participants indicated that education could shield athletes from social pressures and influences that might lead to doping. They also suggested that differences in the level of education, especially between well-developed and less-developed countries, could explain variations in attitudes towards doping. For instance, Athlete 8 detailed their experience with doping prevention workshops and noted the lack of such resources in other countries, suggesting that this discrepancy could contribute to differing attitudes towards doping between countries:

I've gone through numerous workshops about the dangers of doping and like the 100% accountability, everything you put in your body is all your own risk, like there is not a chance of having that in Kenya, I've been to [running camp location in Kenya] and they're not getting that level of education, they're clearly not […] and I think for me that is probably one of the biggest things in why we have an attitude we have in this country and maybe not so much in other countries.

## 4. Discussion

The current study aimed to attain a holistic understanding of athletes’ vulnerability to doping by examining (a) athletes’ perspectives on doping and the value system of those who engage in doping practices, (b) the values that athletes associate with doping and clean sport behaviour, and (c) risk and protective factors that make an individual more or less prone to dope. By focusing on clean athletes, the study provides a unique perspective on doping and those who dope, a viewpoint that might be underrepresented in existing literature. Even though non-dopers form the majority of the athletes who participated in empirical research conducted to date, these studies dominantly focused on factors, motives, and cognitions that lead to doping. Being a non-problematic population, anti-doping rule-compliant clean athletes’ perspectives of doping, dopers and anti-doping have been neglected in the literature. Exceptions to this are recent studies focusing specifically on clean athletes’ personal experiences with doping in their environment and the demands of anti-doping (5, 37, 55). Among other issues, these studies drew attention to how clean athletes feel about sharing training spaces and competing against dopers, including athletes returning to sport after serving an ineligibility period for Anti-Doping Rule Violations. The present study offers insight into how anti-doping-compliant athletes see dopers as individuals and can inform anti-doping policies and practices aiming to protect athletes’ rights to clean sport.

The international group of elite-level athletes in the current study expressed a clear stance against doping, considering it as contradictory to their values. They viewed doping as a result of personal weakness and believed that those who engage in such behaviour do so out of desperation for success, possibly due to a fear of falling behind or in an attempt to recover from injuries. They saw dopers as individuals primarily influenced by external pressures and their internal vulnerabilities. Notably, despite our omission of an explicit definition of doping to the participants, every athlete, without exception, addressed doping as an intentional choice and refrained from discussing inadvertent doping. They presented active and deliberate decision-making processes wherein athletes consciously opted to either utilise or refrain from employing prohibited substances or methods. Furthermore, concerning the ascertained factors that predispose athletes to doping, the participants consistently presupposed that in the instances where athletes had committed anti-doping rule violations, the individuals were fully cognisant of their actions.

The athletes associated doping with negative values, such as a lack of integrity and cheating, while linking clean sports behaviour to positive values, such as hard work, fairness, resilience, and dedication. They regard staying true to personal values, particularly in critical moments, as essential for resisting doping temptations. They also recognised the importance of a strong anti-doping stance and resilience in refusing shortcuts like doping.

Vulnerability to doping can increase due to several factors, including the perception of doping among peers, the pressure to succeed for financial or social reasons, and critical periods such as performance stagnation or injuries. Personality traits, a lack of resilience or an inability to handle pressure can further increase vulnerability. Protective factors included having a strong set of anti-doping values, an established identity outside of sport, supportive relationships (coaches, family, sponsors) that promote clean sport, and education about the dangers and consequences of doping. Athletes who have alternative career plans or academic degrees also seem to be less prone to doping, as they are not entirely dependent on sport for their livelihood or identity.

### 4.1. Athletes condemn doping but understand the circumstances

The participants of this study demonstrated a strong personal stance against doping while recognising the complexity underlying the behaviour of their peers who engage in doping. They acknowledged that doping behaviour is influenced by a mix of personality traits (e.g., integrity), systemic factors (e.g., anti-doping programmes), and situational factors (e.g., their current competitive environment). This is in line with the life-cycle model by Petróczi and Aidman (26) that posits that doping attitudes are the interplay of facilitating and inhibiting personality, systemic, and situational factors. Understanding these factors does not mean that athletes condone doping. Rather, it reflects their recognition of the complexities of the decision-making process around doping and the various influences that can lead an athlete down that path. This nuanced understanding can also make them more empathetic towards those who resort to doping while still maintaining their strong personal stance against the practice. This understanding suggests that they attribute some responsibility to the circumstances surrounding the athlete. However, they also understood that, ultimately, the decision to dope is a personal one. Each athlete must make their own choices, and they believed that athletes who dope have chosen to go against the values of fair competition.

Participants emphasised that doping would contradict their values. They explained that their values protect them from doping as they ascribe high importance to integrity. They had difficulties comprehending how dopers can reconcile their behaviour with their conscience. Some participants suggested that dopers do not experience a value conflict, while others proposed that dopers justify their actions through a process of normalisation [e.g., (56–58)] or moral disengagement (59). They also indicated that the acceptance of doping use happens gradually, starting with small steps and eventually becoming an integral part of training practice.

Despite the protective role of moral values against doping, it appears that justification processes can override the guiding effect of values. This observation supports the findings of Kirby et al. (60). Their qualitative investigation revealed that athletes who had committed doping violations had normalised the use of banned substances up to the point that they did not perceive their behaviour as cheating. Likewise, athletes in previous studies viewed doping as a standard and widespread part of athletic preparation (56). In the present study, athletes speculated that those who engage in doping might initially face a difficult decision, but over time, doping becomes routinised and a regular part of their training regime. This gradual process, often referred to as the “slippery slope”, is believed to contribute to the continued use of performance-enhancing substances. Boardley et al.'s (2014) research corroborates this idea by providing first-hand accounts from athletes who have doped. They described a similar progression, where the initial decision to dope was difficult, but it eventually became a routine part of their training practice.

Participants in the study assumed that dopers might overlook the negative moral consequences of their actions, perceiving doping merely as a means to enhance their performance. This rationalisation aligns with a moral disengagement mechanism known as distortion of consequences. Past research confirmed that dopers tend to downplay or disregard the ramifications of their actions when pursuing personal objectives (61).

Despite the association of moral and self-transcendence values with a lower likelihood of doping (14, 62), these ethical standards do not form an invincible internal control system (63). Justifications can indeed supersede the directive impact of values. Hence, dopers may employ such rationalisations to mitigate the cognitive dissonance caused by conflicts between actions and values (13). As such, if values are to be leveraged to foster clean sports behaviour, understanding athletes’ self-justification processes and equipping them with appropriate counterarguments or strategies are crucial.

### 4.2. No one is born as a doper: acknowledging the dynamic nature of values

Athletes who start doping likely began their sportive careers with the same values as clean athletes, but an interplay of factors led them to shift these priorities. The transition from amateur to professional status is a pivotal event that often facilitates this value change. For example, Mazanov and Huybers (64) showed that non-elite athletes ascribed higher importance to “fun and joy”, whereas elite athletes viewed “dedication and commitment” as more important. It has also been shown that life events may correspond to value change during lifespan (65, 66). Transitioning to elite sports may shift athletes’ values, increasing vulnerability to doping. Sports organisations should acknowledge this and offer support and education during this pivotal phase.

In this study, we identified a specific mechanism related to the dynamic nature of values in the sports environment. Notably, pressure from influential figures in an athlete's life could shape their value system, potentially increasing the likelihood of doping. This pressure can manifest as direct persuasion attempts by coaches, teammates, or others or as indirect influence through environmental cues. Both forms of influence can lead to a long-term shift in value priorities. Furthermore, as athletes often interact with new teams and professional staff throughout their careers, they may adjust their value priorities to align with the group (67). If the elite environment prompts a focus on success and status values, this could increase their vulnerability to doping. These findings were identified through interactions with clean athletes. However, to validate these results and fully understand the dynamics of value shifts in the sports environment, it is essential to conduct similar studies with athletes who have engaged in doping.

### 4.3. Doping as functional tool for performance enhancement

Doping in sports, according to theories like the life-cycle model and Functional Use Theory (9, 26), is primarily seen as a practical tool for enhancing performance rather than a moral issue. This view is particularly pertinent for athletes facing setbacks, as doping serves as a coping mechanism, which has also been supported by Didymus and Backhouse (10), who observed that athletes used permitted and banned substances to cope with stressors, such as injuries or performance pressure. Anti-doping efforts, therefore, need to focus on understanding the performance mindset of athletes (9). They must recognise the high-performance demands of elite sports, including the intense physical and psychological pressures athletes face. These pressures came from a multitude of sources, including the demand for consistent peak performance, quick recovery from injuries, and maintaining a competitive edge. Influences from coaches, teammates, fans, and sponsors also contribute to this pressure. Additionally, it is crucial to comprehend the motivations and thought processes that may lead athletes to view doping as a viable strategy for achieving their goals.

### 4.4. Holistic understanding of doping vulnerability: dynamic and complex influential factors

From the analysis of participants’ statements, it is evident that an athlete's tendency to engage in doping is influenced by a balance of risk factors (which increase vulnerability) and protective factors (which serve as inhibitors), as outlined in the life-cycle model (26). The subsequent section will delve into these vulnerability and inhibiting factors, as identified by the athletes participating in this study.

#### 4.4.1. Vulnerability and risk factors

##### 4.4.1.1. Athletes’ personality

The life-cycle model suggests that certain personality traits can make an athlete more susceptible to doping. This study's participants echoed this, arguing that an individual's character and capacity to handle stress influence their ability to resist doping, especially during periods of vulnerability such as performance frustrations, injuries, or contract pressures. However, these challenges are common in most athletes’ careers. The distinction lies in an individual's psychological resilience and personality traits, which can shield them from succumbing to doping amid external pressures and setbacks. This aligns with previous research emphasising the role of resilience in deterring negative behaviour, even under adverse circumstances (37, 63). Given the perceived vulnerability of young athletes to external pressures, there is a critical need to foster desirable personality traits and teach them self-regulation skills to manage stressors effectively.

##### 4.4.1.2. Systemic factors

Systemic factors, including the performance enhancement culture in teams and broader athletic communities, can increase an athlete's vulnerability to doping (26). This study emphasises the influence of others’ behaviour on individual doping decisions, either through active promotion of illicit substances or passively, by observing others gain competitive advantage from doping. The study participants noted that contact with doping teammates and opponents could potentially influence an athlete's attitude towards doping. This aligns with Connor's (68) concept of the “networked athlete,” emphasising the role of the sporting culture and professional entourage in shaping athletes’ behaviour. This influence can manifest itself either actively, where teammates or staff explicitly promote banned substances, or passively, where athletes observe others gaining an edge through doping. Experienced athletes can especially tempt younger members to use performance-enhancing substances, further perpetuating a doping culture (60, 69).

The participants’ reports highlight the potential adverse effect of not just interacting with doping teammates but also competing against doping opponents. Some athletes use this knowledge as motivation to prove success without banned enhancers, while others may justify doping as a strategic decision to level the playing field. This dynamic nature of doping behaviour underscores the importance of understanding the sporting culture's influence on athletes’ actions.

Moreover, athletes’ professional entourage can significantly impact doping decisions, especially when athletes face emotionally charged situations, high performance pressure, or dependency on coaches and professional staff. This reinforces the need for including athletes’ social networks in anti-doping programmes, recognising the critical role of contextual factors in identifying risky environments and developing targeted prevention strategies.

Additionally, the perception of a low likelihood of being caught for doping undermines the efficacy of detection-based anti-doping strategies. This supports the need for a shift towards a prevention-based approach, as fear of testing positive is often a minor concern for athletes using doping substances (60).

##### 4.4.1.3. Situational factors

Situational factors, which are fluid and constantly changing, significantly influence the interplay between systemic and personality factors in an athlete's decision to engage in doping (26). This study's participants noted that the elite sports environment's characteristics and career-impacting events, such as injuries or economic hardship, could drive athletes towards doping as a coping mechanism. This risk is particularly heightened for older athletes, who may feel an increased sense of urgency. These findings align with previous research that identified transitions to professional status, performance setbacks, or the risk of losing sponsorships as situations that increase doping temptation (10, 60, 61, 69–71). Thus, the present results lend support to the functional use theory, suggesting that when athletes face such challenging situations, they may not perceive doping as cheating but rather as an opportunity to regain their previous performance levels. As such, these situational factors may override personal factors such as cognitive variables, moral beliefs, or motivation, indicating the importance of contextual considerations in understanding and predicting doping behaviours (72).

##### 4.4.1.4. Environmental factors

The life-cycle model (20) underscores that doping does not occur in isolation. The current study's participants echoed this, highlighting the influence of economic and cultural environmental factors. They emphasised the unique pressures faced by athletes from less-developed countries, who often use sport as a means to escape poverty. The financial implications of success in major sporting events for these athletes are profound, leading to higher vulnerability to doping (73, 74). In economically weak environments, the link between values and attitudes can be less straightforward, leading to less value-driven behaviour (75). Participants argued that such athletes, driven by extrinsic motivation, may not place as much emphasis on fairness and rule adherence and, hence, might not perceive doping as a value conflict. Therefore, anti-doping programmes must account for these differing value priorities across countries, challenging the “one size fits all” approach (76). It also underscores the importance of addressing the underlying economic incentives, as simply imparting the moral unacceptability of doping may not be sufficient. This calls for more sustainable solutions.

#### 4.4.2. Protective factors

##### 4.4.2.1. Strong anti-doping values

Participants in the study highlighted a strict moral stance against cheating as an internal deterrent to doping. Upholding values of fairness and respect, along with guilt and shame associated with violating these values, were reported as inhibitory factors. These findings, consistent with earlier research (10, 36, 37, 77), suggest that promoting anti-doping values can be beneficial. Even dopers acknowledged moral concerns as significant deterrents (60, 61), though moral disengagement can occasionally override these values (60, 61, 71). Participants emphasised the importance of being aware of one's value priorities, especially in critical situations. The participants discovered that constantly reinforcing their moral standards facilitated behaviour that aligns with these values. This concept, supported by prior research (78), suggests that having values that are easily accessible and cognitively activated can guide appropriate behaviour (78–80). Conversely, not consciously reflecting on one's values was considered a precursor to cheating and doping. Therefore, dopers may not inherently have different values than clean athletes; they might simply not actively consider their values. Consequently, enhancing athletes’ awareness of their moral values through regular value-based anti-doping programmes could increase the threshold for engaging in transgressive behaviour by reinforcing the connection between actions and feelings of guilt.

##### 4.4.2.2. Identity beyond sport

Participants in this study noted the benefit of having alternatives outside of sports, such as pursuing a professional degree. This broader perspective and financial independence from sports success reduces the pressure from career-affecting events like injuries or performance stagnation. Furthermore, maintaining a social network outside of sports and having support irrespective of sports success was emphasised as vital, as it reassures athletes they are not defined solely by their sports. Prior research supports that athletes less absorbed in their sport are less likely to dope (37, 69). This underscores the need for anti-doping strategies to extend beyond sports, suggesting the expansion and promotion of programmes assisting athletes in pursuing dual careers.

##### 4.4.2.3. Having a supportive and clean environment

Participants asserted that a “clean” environment and anticipated disapproval from significant others act as protective factors against doping. Shared anti-doping values among important people in the athlete's life, along with support that is not contingent on sports success, can bolster the decision to compete clean. Prior research supports the deterrent effect of an anti-doping stance among an athlete's close circle, as well as expected social sanctions for rule-breaking (37, 69). Overbye et al. (36) found that a clean environment's protective effect is even stronger than a doping environment's risk effect. These results highlight the sportive environment's critical role in mitigating doping risks, suggesting that anti-doping programmes should target not only the athletes but also their broader environment to cultivate a community-wide clean sports culture.

##### 4.4.2.4. Type of sport

Participants noted the importance of considering the specific sport when assessing doping risks. Some athletes in this study felt their sport had a low doping risk due to either minimal perceived benefits from doping (e.g., in tactics- or coordination-based sports) or reduced incentives from limited financial rewards. Loland's (sport) vulnerability theory (81) suggests that sports where records are kept - that are, sports where performance is measured in distance, height, weight or time - are vulnerable to disagreeable manipulations that are against the spirit of sport. Indeed, Alaranta et al. (82) also found higher doping temptation in speed and power sports and lower temptation in sports demanding motor skills. Given these variations in doping expectations across sports, anti-doping messages should be customised for each sport.

### 4.5. Practical implications

#### 4.5.1. Better understanding of the dynamics of personal values, performance, and performance enhancement

The personal perceptions highlighted in this study can be used to inform values-based anti-doping education by enhancing the alignment between athletes’ personal values and the values that promote clean sports, as well as identifying and addressing the values that might increase the risk of doping. Values such as fairness, respect for oneself and others, integrity, and adherence to rules are often associated with clean sports. These values can be reinforced through education, encouraging athletes to view these values as integral to their identity as athletes. Some values might increase the risk of doping, such as extreme competitiveness or a win-at-all-costs mentality. While competitiveness is an important aspect of sports, it is crucial to highlight that extreme competitiveness should not compromise ethics and health. Athletes should be encouraged to pursue excellence and strive to win within the boundaries of fairness and respect. Similarly, the win-at-all-costs mentality can be addressed by emphasising the importance of how one achieves success, not just the outcome. In addition, the present results showed that bringing values to conscious understanding could increase the threshold for engaging in transgressive behaviour. Exercises could be included that help athletes identify and articulate their values and how these relate to their behaviour in sport, including their attitudes towards doping. Likewise, educational programmes aiming at moral disengagement and rationalisation, such as dilemma training, may decrease athletes’ likelihood of resorting to doping and foster a stronger value-based stance against doping (83).

Participants in this study showed a strong sense of empathy towards athletes who resort to doping, even though they disagreed with the act itself. This empathy could be strategically incorporated into educational programmes, fostering understanding and compassion among athletes and aiding the process of reintegrating those who have been sanctioned. This approach is congruent with the WADA's International Standard for Education, which emphasises the importance of reintegrating sanctioned athletes (11). Such programmes could involve activities like discussions or role-playing exercises designed to encourage athletes to empathise with the pressures and influences that might lead others to engage in doping.

The participants’ reflections on functional doping use indicate the necessity of endorsing alternative, legitimate methods of performance enhancement. Anti-doping education should underscore the significance of legal and healthy approaches to enhancing performance. These include appropriate nutrition, mental skills training, and various recovery techniques such as adequate sleep, massage therapy, and sauna use. By being informed about these alternatives, athletes might feel less pressured to resort to doping for performance improvement. James (84) provided evidence for this proposal by concluding that the promotion of effective and acceptable alternatives (e.g., functional food) can be a valuable strategy in anti-doping as it might positively affect outcome expectations regarding performance enhancement.

#### 4.5.2. Revised view on athletes’ doping vulnerability

The concept of vulnerability in the context of doping involves systemic, personality, and situational factors that may fluctuate throughout an athlete's career stages (9, 26). This study specifies this concept, indicating that the transition from amateur to elite level represents a critical stage where athletes might begin to perceive doping as acceptable or inevitable. Anti-doping organisations can use this insight to intervene early and prevent the onset of prohibited performance enhancement.

The findings underscored the need for diversified anti-doping strategies tailored to each athlete's career stage, cultural context, and social environment. They highlighted that athletes from lower socioeconomic backgrounds might be more vulnerable due to economic pressures. Hence, there is a need for more country-specific research to develop targeted anti-doping programmes. The study further stressed the role of social context in doping decisions, suggesting that the values and behaviours of coaches, teammates, and family can influence athletes’ choices. Value-based anti-doping education should, therefore, engage all stakeholders to foster a clean sports culture. Importantly, participants in the study did not view dopers as inherently bad but as individuals who succumbed to accumulated risk factors and lacked the personal strength to resist stressors. As such, this suggests that prevention programmes should not just target risk factors but also enhance athletes’ resources to resist the pressure to use doping (e.g., by building resilience and ethical decision-making skills).

Values-based education, when appropriately integrated into early sports development and educational settings, has the potential to significantly augment an individual's capacity for (ethical) decision-making and agency. While it may not act as an impenetrable deterrent for resolute individuals engaging in doping practices, it can serve as a valuable resource for those confronted with challenging situations. Crucially, this approach proves particularly advantageous for athletes committed to upholding clean sport behaviour. Rather than dissuading them from utilising prohibited means, as they have already made a deliberate choice not to do so, it equips them with the requisite skills to cope with the dopogenic environment surrounding them and effectively manage the substantial demands of long-term adherence to anti-doping regulations, often spanning a decade or more. At its core, values-based education primarily fosters the development of self-awareness, empathy, and sense-making abilities within individuals, attributes from which all athletes can derive substantial benefits. The introduction of a system designed to serve and support clean athletes would represent a much-welcomed departure from the predominant “anti-doping” approach commonly seen in anti-doping education. Nevertheless, it is important to note that the provision of information and knowledge remains vital for all athletes to prevent unintentional Anti-Doping Rule Violations. Moreover, when this approach recognises the values attached to performance enhancement, addresses the pressure athletes are under, meets their needs for practical solutions to anti-doping, and is tailored to align with the values held by individual athletes, as opposed to solely emphasising the values endorsed by the anti-doping movement and its affiliated organisations, it stands as an even more effective and desirable strategy (13).

Supplementing the existing view of athletes’ vulnerability (to doping), it is important to draw attention to those who are committed to clean sport behaviour. The participants in the current study pointed out that all athletes start with pure intentions, and only some end up doping. In fact, anti-doping-compliant, clean athletes represent the vast majority of competitors. For them, competing clean is an integral part of their identity as athletes (55, 85). However, in spite of the endeavours made by anti-doping organisations, studies investigating the experiences of clean athletes highlight the negative consequences they face due to their co-existence with dopers, the burden of adhering anti-doping requirements, and the fear of accidental rule violations (5, 55, 85). Still, research specifically focusing on athletes who are committed to clean sports behaviour is scarce. Understanding the reasons, values, and motives of clean athletes is crucial to optimise anti-doping regulations. Therefore, researchers started to call for a shift from a strong focus on catching the dopers to protecting and understanding the desirable behaviour of clean athletes (5, 86). This is key, as the reasons to dope identified in previous studies cannot simply be reversed into protective factors against doping (85). Thus, research should raise clean athletes’ voices to capture their views. Here, it is important to recognise that clean athletes, just like dopers, are not a homogeneous group—neither in terms of their definition of “clean” nor in terms of their exact reasons to compete clean (55, 85, 86). Clean athletes are intrinsically and unquestionably motivated to adhere to anti-doping rules, nevertheless, they face the same challenges as other athletes. Thus, they do not need to be persuaded to compete clean but want support to stay clean and recognition for their efforts to comply with the rules. As of yet, clean athletes’ positive behaviour is often taken for granted and undeservedly overlooked by anti-doping organisations and researchers. Consequently, clean athletes wish that the anti-doping system makes their efforts more visible to others and the system (5, 85, 86).

By placing the spotlight on clean athletes, this study provides a valuable contribution to the current state of anti-doping research and allows a new view of vulnerability. Raising anti-doping-compliant athletes’ voices enriches the understanding of the landscapes of doping and facilitates the design of preventive measures and the incorporation of support for clean athletes. In this way, research can yield benefits for both athletes and anti-doping organisations.

### 4.6. Limitations and future research

The study involves a diverse group of elite athletes from various sports and countries, which enhances the generalisability and richness of the findings, ensuring cultural, societal, and sporting perspectives on doping issues. In contrast to questionnaire-based methods, the design of focus group analysis allows open discussions, stimulates participants to exchange experiences actively, and provides the researcher with in-depth insights. This provides rich information on understanding the motivation to stay clean, values that drive athletes to reject or be vulnerable to doping, and pressures faced by clean athletes and gives insights into the overall culture and acceptance of doping within respective sports.

However, despite the study's breadth in terms of sports types and gender balance, it had less diversity in terms of the participants’ countries of origin. The majority of athletes were from developed nations. Future research should strive to include a more diverse representation from less-privileged regions to fully understand the values and circumstances influencing athletes in these regions. A further limitation of this study is related to the composition of the sample, which most likely consists of anti-doping-compliant, clean athletes. We did not specifically ask athletes to reveal this information, nor doping use vs. abstinence was part of the inclusion criteria, but discussions revealed that most participants had only indirect experiences with doping. Consequently, their perspectives on dopers’ value system and driving factors behind doping are speculative and ego-centrically biased and, therefore, not necessarily reflective of the actual experiences or motivations of athletes who have used doping. Future research focusing specifically on the values and decision-making processes of dopers, compared to clean athletes, would provide a crucial contribution to how values influence decisions related to performance enhancement and doping. Further, the presented work does not cover a comparison between athletes with and without formal anti-doping education. We based this deliberate decision on the conclusion that the presence or absence of this education is likely to exert minimal influence on athletes’ values and perceptions about doping and dopers. This is supported by the fact that the time of data collection preceded the introduction of WADA's International Standards for Education (WADA ISE), a regulatory framework that introduced more comprehensive anti-doping education as a mandatory requirement. As evidenced by Gatterer et al. (87), the majority of National Anti-Doping Organisations (NADOs), primarily conducted information-based anti-doping education with a predominant focus on rules and regulations. Nevertheless, we acknowledge that anti-doping education can have an impact on athlete's inclination to actively support anti-doping measures due to increased anti-doping legitimacy (88). Thus, the investigation of the impact may present a potential avenue for future research.

While the study provides a snapshot of anti-doping-compliant athletes’ motivations, the values guiding their decisions about doping, and the pressures they face, it did not track these aspects through various career stages, particularly the pivotal transition from amateur to elite level, nor did it attempt to make cross-cultural or sports comparisons. Moreover, the presented work does not address how athletes’ reflections on doping and doping users may change after retirement from their sporting careers. This limitation may prevent the study from fully capturing the evolving dynamics and complexities of athletes’ perspectives on doping. Future research employing a longitudinal design could yield a more nuanced understanding of how vulnerability to doping differs in different cultural and sports context shifts across different career phases, thus helping to pinpoint critical moments of vulnerability and informing anti-doping interventions.

## 5. Conclusion

International elite-level athletes possess strong negative perceptions of doping and link it to a violation of their personal values. They viewed doping as a manifestation of personal weakness, desperation for success, or a response to external pressures and personal vulnerabilities. Further, the observation that doping might be justified on the grounds of functionality highlights that anti-doping efforts focusing solely on moral aspects might miss the target if athletes purposefully use doping to expand their athletic performance. Thus, in conclusion, the study suggests that doping is not a simple question of morality but can be a potential vulnerability for any athlete under specific conditions. This may become more pronounced during pivotal career transitions, such as moving from the amateur to the elite level. Furthermore, the results clearly demonstrated that factors such as personality traits, values, sports environment and personal environment present vulnerabilities as well as protection. The specific context in which the decision about initiating and sustaining doping takes place (or not) matters. Thus, developers and facilitators of anti-doping education programmes are advised to not only acknowledge but embrace this important aspect instead of a blanket promotion of the values of sport as the ultimate protective factor.

## Data Availability

The datasets presented in this article are not readily available in order to protect the athletes' identity. The transcripts as a whole contains details and information that could make athletes identifiable. Both our ethical approval and the athletes' consent restrict access to the raw data to the research team, and states that raw data will not be shared beyond the research team. Requests to access the datasets should be directed to Andrea Petroczi, A.Petroczi@kingston.ac.uk.
