# Supplementary material for: Context matters: athletes’ perception of dopers’ values, actions and vulnerabilities
Source: Front Sports Act Living. 2023 Dec 15;5:1229679. doi: 10.3389/fspor.2023.1229679 (PMC10757837; doi:10.3389/fspor.2023.1229679)
Supplement: Supplementary file 1 [file Datasheet1.pdf]

Context matters: Clean athletes' perception of dopers' values, actions and vulnerabilities

Annalena Veltmaat, Dennis Dreiskaemper, Sebastian Brueckner, Dmitriy Bondarev, Andrew Heyes, Vassilis Barkoukis, Anne-Marie Elbe, Lambros Lazuras, Alessandra De Maria, Arnaldo Zelli Andrea Petróczi

Supplementary Material

Supplementary Material 1: Demographics of the FG participants

ID	Focus Group	Nationality	Status	Gender	Age	Sport
Athlete 1	National	Germany	active	male	21	Biathlon
Athlete 2	National	Germany	retired	female	29	Floorball
Athlete 3	National	Germany	retired	male	19	Luge
Athlete 4	National	Germany	retired	male	23	Athletics
Athlete 5	National	Germany	active	female	27	Judo
Athlete 6	National	UK	active	female	23	Football
Athlete 7	National	UK	active	male	31	Para Powerlifting
Athlete 8	National	UK	active	male	26	Athletics - distance
Athlete 9	National	UK	active	male	19	Athletics - 800m
Athlete 10	National	UK	active	male	24	Athletics - 800/1500
Athlete 11	National	UK	active	female	21	Field hockey
Athlete 12	National	UK	active	male	24	Athletics - steeplechase
Athlete 13	National	Italy	active	male	20	Athletics- throwing
Athlete 14	National	Italy	active	male	21	Kickboxing
Athlete 15	National	Italy	active	female	25	Tennis
Athlete 16	National	Italy	retired	female	27	Synchronised ice skating
Athlete 17	National	Italy	active	female	21	Sailing
Athlete 18	National	Italy	active	male	20	Kickbox/Taekwondo, ITF
Athlete 19	National	Greece	active	male	21	Decathlon
Athlete 20	National	Greece	active	male	21	Decathlon
Athlete 21	National	Greece	active	female	26	Sailing
Athlete 22	National	Greece	active	female	25	Sailing
Athlete 23	National	Greece	active	male	18	Handball
Athlete 24	National	Greece	active	male	38	Basketball
Athlete 25	National	Greece	active	male	20	Athletics - Sprint
Athlete 26	National	Greece	active	male	24	Taekwondo/martial sports
Athlete 27	National	Greece	active	male	40	Archery
Athlete 28	National	Greece	active	male	18	Football
Athlete 29	National	Russia	active	female	22	Athletics
Athlete 30	National	Russia	active	female	19	Athletics
Athlete 31	National	Russia	active	male	19	Football
Athlete 32	National	Russia	active	male	18	Weightlifting
Athlete 33	National	Russia	active	male	21	Wrestling
Athlete 34	National	Russia	active	female	18	Volleyball
Athlete 35	National	Russia	active	male	17	Football
Athlete 36	International	Italy	retired	female	34	Athletics - throwing

Athlete 37	International	Greece	active	female	23	Handball
Athlete 38	International	UK	active	male	28	Athletics
Athlete 39	International	Italy	retired	female	33	Athletics - sprint
Athlete 40	International	Germany	retired	male	32	Ice speed skating
Athlete 41	International	Greece	retired	male	40	Open Water Swimming
Athlete 42	International	UK	active	female	27	Mixed Martial Arts
Athlete 43	International	Russia	retired	male	25	Bodybuilding
Athlete 44	International	Russia	retired	female	22	Tennis
Athlete 45	International	UK	active	female	23	Weightlifting
Athlete 46	International	Greece	active	male	33	Athletics - high jump
Athlete 47	International	Germany	retired	male	32	Cycling - road racing
Athlete 48	International	Grenada	active	male	21	Boxing
Athlete 49	International	Denmark	active	female	32	Triathlon
Athlete 50	International	Turkey	active	male	20	Artistic Gymnastics
Athlete 51	International	Spain	active	female	34	Athletics- distance
Athlete 52	International	Ireland	active	male	48	Paracanoeing
Athlete 53	International	Turkey	retired	male	31	Wrestling
Athlete 54	International	Argentina	active	female	30	Handball
Athlete 55	International	Austria	active	female	33	Skeleton
Athlete 56	International	UK	active	male		Triathlon
Athlete 57	International	UK	active	male	20	Weightlifting
Athlete 58	International	Serbia	active	female	35	Shooting
Athlete 59	International	Ireland	active	male	36	Athletics – distance
Athlete 60	International	UK	active	male	40	Athletics - distance

Supplementary material 2: Definitions

Themes and subthemes		Definition
1. Clean athletes' personal stance on doping	Within this theme, participants' personal stance on doping was examined to better understand why they refused to engage in doping and to classify their statements about the value systems and thought processes of doping-users.	
	**1.1 Doping is Unacceptable**	When discussing the value system of dopers, the athletes also state their personal opinion about doping. They argue that (for them) doping is mainly unacceptable because it is cheating of self and others and breaks the bond of equality between athletes.
	**1.2 Demonstrating Understanding for Doping Users**	Participants clearly distance themselves from doping behaviour. Yet they showed understanding toward athletes who used doping. As much as they despise the idea of doping, they have sympathy for some athletes who might had no other choice but to dope.
	**1.3 Doping is Not a Solo Act**	Athletes explain that the decision to dope is often not a solo act, i.e., there must be a system or people behind them that are actively or passively dragging them to use doping. Participants state that especially at a young age, athletes are (emotionally) dependent on their close contact persons (e.g. the coach, teammates) and may be easily persuaded by their input.
2. Dopers in the Eyes of the Clean Athletes	Clean athletes' statements were analysed to investigate their perception of dopers. By doing so, similarities and differences between clean athletes' values and the perceived value priorities of dopers can be identified, and the significance of values for the doping decision can be examined.<br><br>Within this theme, two contradictive views were identified: 1) Dopers are not like us, 2) Dopers are no evil, they are more like you and me than we think.	
	**2.1 "Everybody is so different" – Dopers Cannot Have the Same Value Priorities**	Athletes point out the differences between doping users and non-users. As they view having the same values as them and doping as incompatible, they argue that they must prioritise different values.

- Dopers Do Not Experience a Value Conflict	Participants assume that dopers view their behaviour as justified ("everyone does it"). They explain that for dopers, the practice of illegal performance enhancement does not contradict their value priorities, or they simply hold a "don't care"- attitude. Hence, they surmise that dopers do not experience a value conflict.
- The Multifaceted Value Spectrum of Dopers	As their personal values conflict with cheating behaviour (incl. doping), clean athletes expect dopers to hold different value priorities. Moreover, participants acknowledge that the value priorities of dopers cannot be generalized as there is no uniform set of values that all athletes hold, dopers included.
- Dopers Prioritise Less Desirable or Less Social Values	Participants expect athletes who decide to dope to be guided by less social/less desirable values, i.e., they prioritise money, fame, and success over honesty, fairness, dedication, and/or respect
**2.2 A Doper is What a Doper does**	Despite the fact that the interviewed athletes stated that they would never dope, some of them showed a certain level of understanding for other athletes' decision to use doping. They explain, that rule breakers are not necessarily bad persons ("they are just like us") but that the decision to dope might be the result of the circumstances these athletes had to face during their career.
- Dopers Started With Pure Intentions and Changed their Values over Time	The participants claim that no athlete starts sports with the predetermined goal to dope. Instead, they shared the same values as clean athletes when they started but influential factors caused them to change/loose/ abandon their priorities at some point in their career.
- Dopers Are No Evil and Intend No Harm	Participants argued that dopers do not use doping to harm but to improve their athletic performance or living conditions (i.e., doping is viewed as a functional behaviour rather than a deviant act).

	- Doping is Not Value-Driven	Athletes argue that values might not be the decisive factors that explain doping behaviour.
3. Doping Vulnerability is a balance	This theme includes statements that indicate that athletes' doping vulnerability emerges from the balance of the risk factors (R) they are exposed to and the protective factors (P) that shield them from the temptation to engage in illicit performance enhancement.	
	**3.1 Sport Environment**	
	- High Pressure of Elite Sport (R)	Being involved in professional sport is highly demanding, both physically and mentally. Not being able to withstand the enormous pressure in professional sports might cause some athletes to take doping to improve their chances of success. Among other things, this refers to the financial pressure or the pressure to meet the expectations of others (coaches, sponsors, fans etc.).
	- Seeing Others Doping (R)	Observing others gaining a competitive advantage from doping use might increase the temptation to engage in it as well.
	- Low Risk Types of Sport (P)	Those in low-risk sport for doping consider themselves lucky as they view the incentives to dope as low (e.g., tactical sports or sports with low financial rewards).
	**3.2 Personal Environment**	
	- Feeling Safe to Dope (R)	The temptation to dope increases when observing others benefitting from doping and getting better. This increases even further when athletes perceive the risk of getting caught as low.
	-Supportive Environment (P)	Athletes argue that being surrounded and supported by people who share the same values (also regarding doping) helps them to act in line with their values without feeling pressured to dope.
	**3.3 Functions vs. values**	
	- Performance Enhancement During Critical Periods (R)	Participants assume that some athletes might dope because they fear to "fall behind" their competitors (e.g., due to age, injury etc.). When experiencing these setbacks the temptation to dope is especially high as it might allow the

	athletes to improve (or to get back to their previous performance level).
- Being Conscious of One's Values (P)	Athletes classify having strong anti-doping values and being consciously aware of one's value priorities, especially when being confronted with critical situations, as a protective factor.
**3.4 Personality and Identity**	
- Personality and Weaknesses (R)	Athletes argue that dopers might share the same values, but it's rather a matter of personality and weakness (e.g., lack of resilience, being easily influenced, giving in easily) than a matter of values.
- Not Just an Athlete (P)	Depending on the reason why someone engages in sport also the importance of being successful varies. Whereas some athletes only do it for fun, health, and their personal satisfaction, other athletes (from certain (less-developed) countries) use sport as a tool to escape poverty and/or to improve their living conditions. For the latter doping is a justified/necessary mean to achieve their goal.<br>Hence, the exclusivity of sport as the only career option increases the pressure to perform well so athletes who don't have any other options besides sport might feel the need to do whatever it takes to stay in sports. Yet, athletes argue that pursuing a dual career (i.e., not being dependent on success in sports) helps them to reduce the stress/temptation to dope and to deal with setbacks.
**3.5 Education for All (P)**	Receiving anti-doping education serves as protective factor as it increases the awareness about the consequences of doping.
